# Improving Vulnerability Management for Security-by-Design of Medical Devices

**DOI:** 10.3390/s25144418

**Published:** 2025-07-16

**Authors:** Emanuele Raso, Francesca Nanni, Francesco Lestini, Lorenzo Bracciale, Giorgia Panico, Giuseppe Bianchi, Giancarlo Orengo, Gaetano Marrocco, Pierpaolo Loreti

**Affiliations:** 1 Department of Electronic Engineering, University of Rome Tor Vergata, 00133 Rome, Italy; lorenzo.bracciale@uniroma2.it (L.B.); giorgia.panico@uniroma2.it (G.P.); giuseppe.bianchi@uniroma2.it (G.B.); orengo@ing.uniroma2.it (G.O.); pierpaolo.loreti@uniroma2.it (P.L.); 2 Department of Civil Engineering and Computer Science Engineering, University of Rome Tor Vergata, 00133 Rome, Italy; francesca.nanni@uniroma2.it (F.N.); francesco.lestini@uniroma2.it (F.L.); gaetano.marrocco@uniroma2.it (G.M.)

**Keywords:** cybersecurity, medical devices, security-by-design, CTI, MISP

## Abstract

The healthcare industry is witnessing a rapid rise in the adoption of wearable and implantable medical devices, including advanced electrochemical sensors and other smart diagnostic technologies. These devices are increasingly used to enable real-time monitoring of physiological parameters, allowing for faster diagnosis and more personalized care plans. Their growing presence reflects a broader shift toward smart connected healthcare systems aimed at delivering immediate and actionable insights to both patients and medical professionals. At the same time, the healthcare industry is increasingly targeted by cyberattacks, primarily due to the high value of medical information; in addition, the growing integration of ICT technologies into medical devices has introduced new vulnerabilities that were previously absent in this sector. To mitigate these risks, new international guidelines advocate the adoption of best practices for secure software development, emphasizing a *security-by-design* approach in the design and implementation of such devices. However, the vast and fragmented nature of the information required to effectively support these development processes poses a challenge for the numerous stakeholders involved. In this paper, we demonstrate how key features of the Malware Information Sharing Platform (MISP) can be leveraged to systematically collect and structure vulnerability-related information for medical devices. We propose tailored structures, objects, and taxonomies specific to medical devices, facilitating a standardized data representation that enhances the security-by-design development of these devices.

## 1. Introduction

The healthcare industry is witnessing a rapid rise in the adoption of wearable and implantable medical devices, including advanced electrochemical sensors and a wide range of other smart diagnostic technologies. These innovations are increasingly being integrated into next-generation medical systems to enable real-time and continuous monitoring of critical physiological parameters such as glucose levels, heart rate, body temperature, respiration, and even neural activity. Examples include optical sensors for oxygen saturation and blood flow, bioimpedance sensors for hydration and respiratory tracking, electrophysiological sensors such as ECG and EMG for cardiovascular and muscular activity, and piezoelectric sensors for detecting pressure or mechanical strain within tissues.

The utility of these sensors lies not only in their accuracy and miniaturization but also in their compatibility with biocompatible materials that allow for safe long-term use in or on the human body. Their integration into smart and connected platforms—commonly referred to as medical Internet of Things (IoT) devices—marks a paradigm shift toward proactive data-driven healthcare. These systems are capable of generating large volumes of health-related data in real time to support timely diagnoses, early warning systems, personalized treatment plans, and more effective patient follow-up.

Moreover, the ability of these devices to provide remote, high-frequency, and context-aware monitoring enhances the responsiveness of healthcare professionals, enabling more informed and rapid medical decisions. This is particularly crucial in chronic care, post-operative recovery, and emergency medicine. By improving interoperability, safety, and patient engagement, smart wearable and implantable devices are becoming essential components in modern healthcare ecosystems, fostering better communication between patients and clinicians while also supporting the broader goals of preventive medicine and digital health transformation. However, as emphasized in [1], the integration of such connected technologies must be accompanied by robust and well-defined security frameworks, without which the confidentiality, integrity, and availability of sensitive medical data can be compromised. Due to their continuous data collection and wireless communication, medical IoT devices increase the system’s attack surfaces, and as such require explicit policy-driven mechanisms to control access, authenticate users, and ensure auditability. All of these principles are critical for protecting both patients and healthcare infrastructure within the evolving digital landscape.

The healthcare sector has recently witnessed a surge in cyberattacks. Between 1 January 2018 and 30 September 2023, the U.S. Department of Health and Human Services Office for Civil Rights reported a 239% increase in hacking-related data breaches and a 278% rise in ransomware incidents [2]. Additionally, 92% of healthcare organizations were affected by cyberattacks in 2024, up from 88% in the previous year [3]. Compromised healthcare information can be up to fifty times more valuable than financial data, with complete medical records fetching as much as USD 1000. According to [4], malware remains the most prevalent attack vector (35%), with exploitation of known vulnerabilities surging from 11% in 2022 to 24% in 2023. This trend underscores the urgent need for enhanced cybersecurity measures and greater preparedness across the healthcare industry.

The research community has responded to these security challenges by developing comprehensive frameworks for securing medical devices, including recent work on secure IoT implementations for healthcare applications [5].

Modern Implantable Medical Devices (IMDs) introduce additional security concerns due to their wireless capabilities [6,7]. Threats such as unauthorized access, eavesdropping, message replay, Man-in-the-Middle (MITM) attacks, and impersonation can compromise patient safety. Attackers may manipulate device settings, disrupt treatment, or even shut down devices remotely. Denial of Service (DoS) attacks can pose life-threatening risks by interrupting communication and draining battery power. Additionally, vulnerabilities in IMD firmware and remote attacks targeting network-connected medical devices can serve as entry points to hospital networks, endangering both patient data and critical infrastructure [8,9].

As medical data become increasingly susceptible to breaches, addressing security challenges and mitigating emerging threats in healthcare organizations is crucial. *Medical device security* aims to ensure that devices function correctly even under malicious attacks. This principle aligns with McGraw’s definition of software security as “building software to be secure from the ground up, so that it continues to function correctly under malicious attack” [10]. This encompasses hardware and software protections against both intentional and unintentional threats [11].

Healthcare systems require strict security and safety standards to protect patient data and maintain system integrity. However, resource constraints—especially in IMDs—render traditional security measures impractical. Limited power and storage capacity prevent the deployment of antivirus software, as it could rapidly deplete battery life and introduce new vulnerabilities. Additionally, memory constraints hinder the use of standard security software and complicate timely vulnerability patching, as updates necessitate re-certification.

In this complex landscape, adopting the *security-by-design* paradigm is imperative. Unlike reactive approaches that address vulnerabilities post-exploitation, this proactive methodology integrates security measures from the initial stages of system design, ensuring robust protection against potential threats [12].

Unfortunately, current regulations in many jurisdictions often emphasize general recommendations for medical device cybersecurity rather than specifying mandatory security requirements; for example, the U.S. Food and Drug Administration (FDA) provides cybersecurity guidance documents that describe recommended practices, but these are not legally binding requirements except where they intersect with broader quality system regulations [13]. Similarly, while the European Union’s Medical Device Regulation (MDR) and In Vitro Diagnostic Regulation (IVDR) establish general safety and performance requirements, they do not lay out concrete cybersecurity obligations; instead, the MDCG 2019-16 guidance provides only non-binding recommendations [14,15,16]. In the UK, the Medicines and Healthcare products Regulatory Agency (MHRA) issues guidance aligned with international norms, framing cybersecurity largely in terms of best practices [17]. India’s Central Drugs Standard Control Organization (CDSCO) has limited prescriptive detail on cybersecurity, relying on adherence to international standards [18]. While compliance is enforced by notified bodies and regulators, much of the cybersecurity content is advisory rather than prescriptive, creating an environment where manufacturers may lack clear and actionable obligations.

Moreover, post-market solutions often struggle to accommodate device constraints such as limited computational resources, rendering security patching impractical or even infeasible for certain devices [11]. This persistent gap is compounded by the prevalent lack of specialized cybersecurity training and the scarcity of design-support tools specifically tailored to the unique constraints of medical devices [19].

A major challenge in developing such tools and training programs is the heterogeneity and sheer volume of relevant security information. Security resources span diverse formats from regulatory texts and standards to technical threat databases, which can complicate effective retrieval, analysis, and integration. These data integration barriers significantly raise the complexity and cost of developing robust cybersecurity support tools for medical device manufacturers.

### Our Contribution

To enhance awareness of cybersecurity issues in medical devices, this work proposes a methodology for streamlining the communication of critical information to development and design teams in the medical industry. Our approach introduces a Cyber Threat Information Sharing (CTIS) platform built on the open-source Malware Information Sharing Platform (MISP) threat sharing system. This platform aggregates data from various sources into a centralized hub, improving accessibility and facilitating collaboration among different stakeholders.

By defining custom structures and taxonomies specifically tailored to medical devices, we establish a standardized data representation format that enhances usability. In particular, we introduce two custom data structures: one for Industrial Control Systems Medical Advisory (ICSMA), and another for vulnerable medical devices. In our opinion, the latter is especially valuable, since it enables the creation of content that is highly relevant to platform users.

To summarize, our contributions are as follows:

- An MISP-based CTIS platform for medical devices.
- A custom taxonomy for representing vulnerable medical devices.
- Integration of this taxonomy into MISP’s IoC events to enhance information usability.

The remainder of this paper is organized as follows. Section 2.1 presents software and hardware methodologies applicable to device development. Section 3 presents the most relevant vulnerability sources chosen. To address the heterogeneity of their provided information, Section 4 introduces our custom taxonomy for representing vulnerable medical devices and details MISP as the chosen platform for data collection and utilization. Section 5 explains how we tailored MISP structures to our needs, while Section 6 highlights some interesting use cases. Finally, conclusions are drawn at the end of the paper.

## 2. Background

### 2.1. Device Development Methodologies

Adopting a security-by-design approach is crucial to minimizing device vulnerabilities by embedding security considerations throughout the entire device lifecycle, from initial design to deployment and maintenance [20]. The implementation of rigorous security practices at all stages encompasses both software and hardware components to facilitate proactive threat mitigation, thereby reducing the risk of external attacks and operational failures.

#### 2.1.1. Secure Software Development

Secure Software Development (SSD) [21] involves the integration of security principles and best practices throughout the entire software development life cycle (SDLC). This methodology ensures that security is an integral consideration at every phase, from planning and design through to implementation, verification, and maintenance. The adoption of *Secure Software Development Frameworks* (SSDFs) such as NIST’s SSDF [22], the OWASP Software Assurance Maturity Model (SAMM) [23], Microsoft’s Security Development Lifecycle (SDL) [24], or compliance with ISO/IEC 27034 for application security [25] is particularly critical. Organizations are often required to establish a *Secure Development Policy*, especially those aiming for compliance with standards such as SOC 2 Type 2 [26] or ISO 27001 [27]. Such policies provide structured guidelines and procedures to minimize vulnerabilities across the SDLC. Furthermore, they offer a framework for evaluating and demonstrating security at each development stage, integrating risk management strategies to ensure comprehensive protection.

The use of SDLC as a solution to address cybersecurity challenges in life-saving devices was previously proposed in [28]. The author emphasized that following secure SDLC processes can mitigate threats and vulnerabilities, thereby ensuring the safety of critical systems and devices vital to human life.

##### Threat Modeling and Security Requirements Planning

SSD prioritizes proactive risk identification and mitigation, employing techniques such as threat modeling and risk assessment. A core aspect is the design of secure architectures that minimize the attack surface and implement defense-in-depth strategies.

##### Secure Coding

Secure coding refers to the adoption of programming practices that prioritize software security by minimizing vulnerabilities, protecting sensitive data, and ensuring resilience against attacks. Secure coding employs techniques such as input validation, proper error handling, and secure data storage to mitigate common security risks. To maintain robust security measures, developers must remain informed about emerging vulnerabilities, attack vectors, and evolving best practices. Furthermore, secure coding necessitates the implementation of appropriate cryptographic algorithms and secure communication protocols to safeguard data both in transit and at rest. A key principle of secure coding is the *principle of least privilege*, which dictates that software components should only possess the minimum access rights necessary to perform their functions. To promote consistent security practices, secure coding adheres to established standards and guidelines, such as the SEI CERT Coding Standards [29] and the CERT Secure Coding Practices [30]. In addition, resources such as the OWASP Secure Coding Practices [31] provide practical checklists and recommendations, while the OWASP Top 10 [32] identifies the most critical web application security risks, helping developers to prioritize threat mitigation. By following these best practices and resources, developers can enhance software security, mitigate risks, and build trustworthy applications that resist malicious activities.

##### Code Review, Security Testing and Secure Configuration Management

Effective SSD incorporates rigorous code review practices involving regular examination of code for potential security issues and ensuring adherence to secure coding standards. Complementing this, security testing is vital, including the utilization of methodologies such as penetration testing, vulnerability scanning, and detailed code analysis to detect and remediate security flaws. Furthermore, secure configuration management is essential for implementing and maintaining secure settings for software systems and their operational environments.

##### Security Awareness Training, Regular Updates and Patches, Vulnerability Management and Incident Response

A comprehensive SSD strategy also encompasses several ongoing critical processes. Security awareness training for developers is fundamental, enhancing their ability to implement secure coding practices and stay informed about emerging threats. The consistent application of regular updates and patches is crucial for addressing known vulnerabilities and protecting against new threats. Effective vulnerability management involves a continuous process of identifying, assessing, and remediating security weaknesses. Finally, a well-defined incident response plan must be in place, outlining procedures for containment, eradication, and recovery in the event of a security breach. These critical processes are supported by secure deployment practices such as proper server and network configuration, secure update mechanisms, and robust access controls, and are maintained through continuous security monitoring post-deployment to ensure ongoing protection.

#### 2.1.2. Hardware Security

To ensure the security and integrity of medical devices, robust hardware security measures must be implemented. While network-based attacks are well documented, the hardware itself can also serve as an attack vector, especially when an attacker has physical access to the device [33,34]. This risk is significant for implantable or wearable medical devices, which may be exposed in public spaces such as hospitals, stadiums, or transportation hubs.

Medical devices should be designed to resist unauthorized physical access through tamper-evident enclosures, anti-tampering sensors, and protective coatings that hinder hardware modification. Electromagnetic shielding and power isolation reduce susceptibility to Electromagnetic Interference (EMI) and Power Side-Channel (PSC) attacks [35,36]. Secure boot mechanisms, firmware integrity checks, and cryptographic authentication protocols help to prevent unauthorized code execution and data extraction. Strong authentication methods such as multi-factor authentication and role-based access control, ensure that only authorized entities can interact with the device.

Despite these measures, certain attacks remain a concern. EMI-based attacks can induce voltage or current fluctuations in analog sensors, potentially causing unintended or dangerous device behaviors such as accidental pacemaker discharges [35]. Out-of-band signal injection attacks exploit the physical response of a device to unintended signal frequencies, interfering with its normal function [37]. PSC attacks allow attackers to extract cryptographic keys or sensitive data by analyzing electromagnetic emissions or power consumption patterns [36]. While these attacks are particularly effective against large on-site medical equipment such as MRI or X-ray machines, they are less feasible for implanted devices due to their proximity requirements.

Hardware security is essential for protecting medical devices from both cyber and physical threats. Implementing tamper resistance, isolation, secure firmware management, and strong authentication reduces the risk of attacks. Although hardware attacks such as EMI manipulation and PSC remain a concern, well-designed security frameworks significantly mitigate potential exploits, ensuring patient safety and data integrity.

## 3. Data Sources

A proper medical device development process that incorporates security-by-design requires a thorough understanding of the existing threats and attack surfaces specific to the device in question. Knowledge of known vulnerabilities present in similar devices is also crucial. The sources of this information are diverse and varied. In particular, we believe that the three most relevant sources are *Common Vulnerabilities and Exposures* (CVE), *Industrial Control Systems Medical Advisory* (ICSMA), and the scientific literature.

### 3.1. CVE

Common Vulnerabilities and Exposures (CVE) is a publicly accessible dictionary of known cybersecurity vulnerabilities and exposures, each identified by a unique CVE ID. These IDs are used to track and coordinate vulnerability information across different security tools and services.

CVE IDs are assigned by *CVE Numbering Authorities* (CNAs) [38], which include security researchers, software vendors, and government agencies authorized by the CVE Program [39]. While CVEs are primarily associated with software vulnerabilities, they can also be assigned to hardware, firmware, and other system components.

When a CVE is assigned, a corresponding record is created in the CVE database. Each record contains details about the vulnerability, including its description, impact, and remediation steps. Additionally, CVEs are used to generate *Common Vulnerability Scoring System* (CVSS) scores, which quantify the severity of vulnerabilities. These scores help security professionals to prioritize remediation efforts and inform their risk management decisions.

CVEs play an important role in vulnerability management by providing a standardized framework for describing and classifying vulnerabilities. This standardization enhances communication and collaboration among security professionals, making vulnerability information more accessible and ultimately improving the security of software and systems.

### 3.2. ICSMA

An Industrial Control Systems (ICS) Medical Advisory (ICSMA) is a security advisory issued by the *Cybersecurity and Infrastructure Security Agency* (CISA) [40], to alert healthcare organizations about vulnerabilities and exploits that could affect ICS medical devices such as ventilators, anesthesia machines, and dialysis machines.

ICSMAs provide healthcare organizations with essential information, including details on identified vulnerabilities and exploits, the specific medical devices and manufacturers affected, and the potential impact on device functionality. Additionally, they offer recommended mitigation strategies to safeguard medical devices along with references to further resources such as vendor security patches and technical guidance.

As a vital resource for healthcare organizations, ICSMAs help strengthen the security of medical devices. By regularly reviewing these advisories and implementing appropriate mitigation measures, healthcare organizations can reduce the risk of cyberattacks and enhance patient safety.

### 3.3. Scientific Literature

CVEs and ICSMAs are inherently valuable sources of certified information. An additional valuable contribution may come from the scientific literature, which offers complementary insights into documented CVEs and may provide critical information relevant to the secure development of medical devices.

## 4. Data Classification and Sharing

As discussed in Section 3, the sources of information relevant to a secure medical device development process are diverse and heterogeneous. When this information is structured, each source typically employs a different data representation. Consequently, accessing all available information requires users to be familiar with multiple “languages”. These users include not only individuals involved in the device development process but also automated systems responsible for verifying security.

For these reasons, it is crucial to adopt a standardized format for data representation. Such a format harmonizes input from various sources and facilitates interoperability between information repositories and medical device development and testing systems. Specifically, a common format should enable different entities to extend its content, incorporating new information from diverse sources to enhance its utility.

### 4.1. Medical Device Taxonomy

A taxonomy has been defined to classify medical devices and explicitly represent information regarding their security and vulnerabilities. This taxonomy is promoted by the *Cyber4Health* Observatory on Cyber and Physical Vulnerabilities in Medical Devices [41], and profiles devices based on the eight key criteria reported in Table 1.

- *Body District*—The specific body part the device is intended to affect (e.g., heart, stomach).
- *Product Type*—The category of medical device based on function (e.g., insulin pumps, pacemakers).
- *Device Type*—Classification into *wearables* (e.g., insulin pumps), *implantables* (e.g., pacemakers), *smartwatches*, or *on-site* devices.
- *Year*—The year in which the vulnerability was discovered.
- *Attack Type*—The nature of potential attacks the device is susceptible to (e.g., cyber, physical).
- *Vulnerability Type*—The type of detected vulnerability (e.g., lack of encryption, lack of authentication).
- *Vulnerability Severity*—The CVSS score assessing the severity of the vulnerability.
- *Risk Class*—The device hazard classification, either *I*, *IIa*, *IIb*, or *III*.

This taxonomy is fundamental in defining the data format used in our solution, which is detailed in Section 5.

### 4.2. European Medical Device Nomenclature

Article 26 of Regulation (EU) 2017/745 on medical devices [14] and Article 23 of Regulation (EU) 2017/746 on in vitro diagnostic medical devices [15] mandate that the European Commission provide, free of charge, a nomenclature of medical devices recognized at the international level [42]. This list is open to update proposals from various stakeholders, including competent authorities, notified bodies, the World Health Organization (WHO), trade associations, manufacturers, authorized representatives, importers, and distributors. Proposals can be submitted throughout the year following a dedicated procedure; at the end of each year, a revised version of the list is published. Manufacturers use this nomenclature for registering medical devices in the EUDAMED database [43].

**Figure 1:**
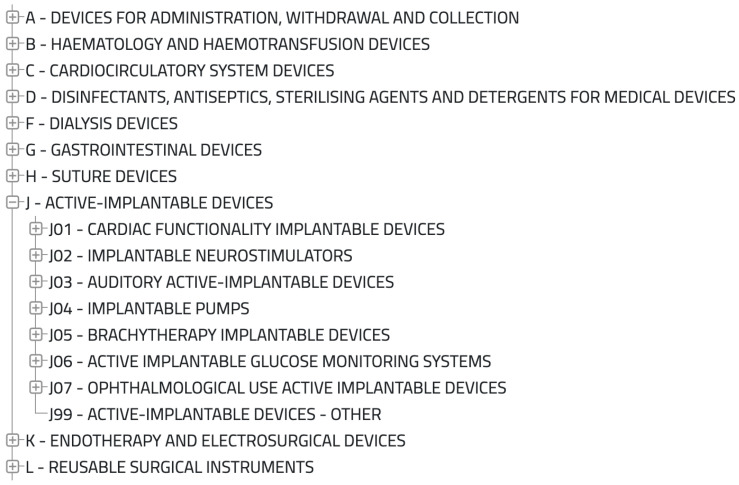
Subset of the EMDN.

As illustrated in Figure 1, the EMDN follows a hierarchical structure, allowing for a detailed classification of medical devices based on their specific characteristics.

Thus, the EMDN serves as a valuable, internationally recognized, and standardized classification tool that can be leveraged to refine the data format used in our solution.

### 4.3. Data Sharing Tools: MISP

The *Malware Information Sharing Platform* (MISP) [44] threat sharing tool is an open-source threat intelligence platform designed to enhance cybersecurity efforts by facilitating the exchange of threat intelligence among organizations and communities worldwide. Its primary goal is to strengthen collective defense against cyber threats by enabling structured and collaborative threat information sharing.

MISP serves as a central repository where organizations can share and analyze a wide range of threat intelligence data, including *indicators of compromise* (IoCs), malware samples, attack techniques, and *tactics, techniques, and procedures* (TTPs). By leveraging shared intelligence, organizations can proactively enhance their security posture.

A key feature of MISP is its standardized data format, which ensures consistency and interoperability across different organizations and platforms. The platform is highly extensible and integrates seamlessly with various cybersecurity tools, including Security Information and Event Management (SIEM) systems, Intrusion Detection/Prevention Systems (IDS/IPS), and firewalls. It supports diverse data import and export formats, making it adaptable to different security infrastructures.

One of MISP’s core functionalities is IoC management, allowing organizations to collect, update, and share IoCs in real time. It aggregates threat intelligence from multiple sources, including commercial, open-source, and community-contributed feeds, providing up-to-date insights into emerging threats.

MISP fosters collaboration by enabling the creation of private or community-specific threat-sharing groups, allowing organizations to share intelligence within trusted networks. Additionally, the platform offers powerful tools for threat analysis and visualization, helping analysts identify correlations, uncover hidden relationships, and detect patterns that might not be evident when analyzing individual indicators.

In incident response, MISP provides a structured framework for documenting and handling incidents. Analysts can attach threat intelligence data to incidents, aiding in risk assessment and mitigation. The platform also emphasizes privacy and data-sharing controls, allowing organizations to define granular access permissions to ensure compliance with legal and regulatory requirements.

MISP’s development is continuously supported by an active community of cybersecurity professionals, fostering innovation and ensuring its relevance in the ever-evolving cyber threat landscape.

## 5. Medical MISP

**Figure 2:**
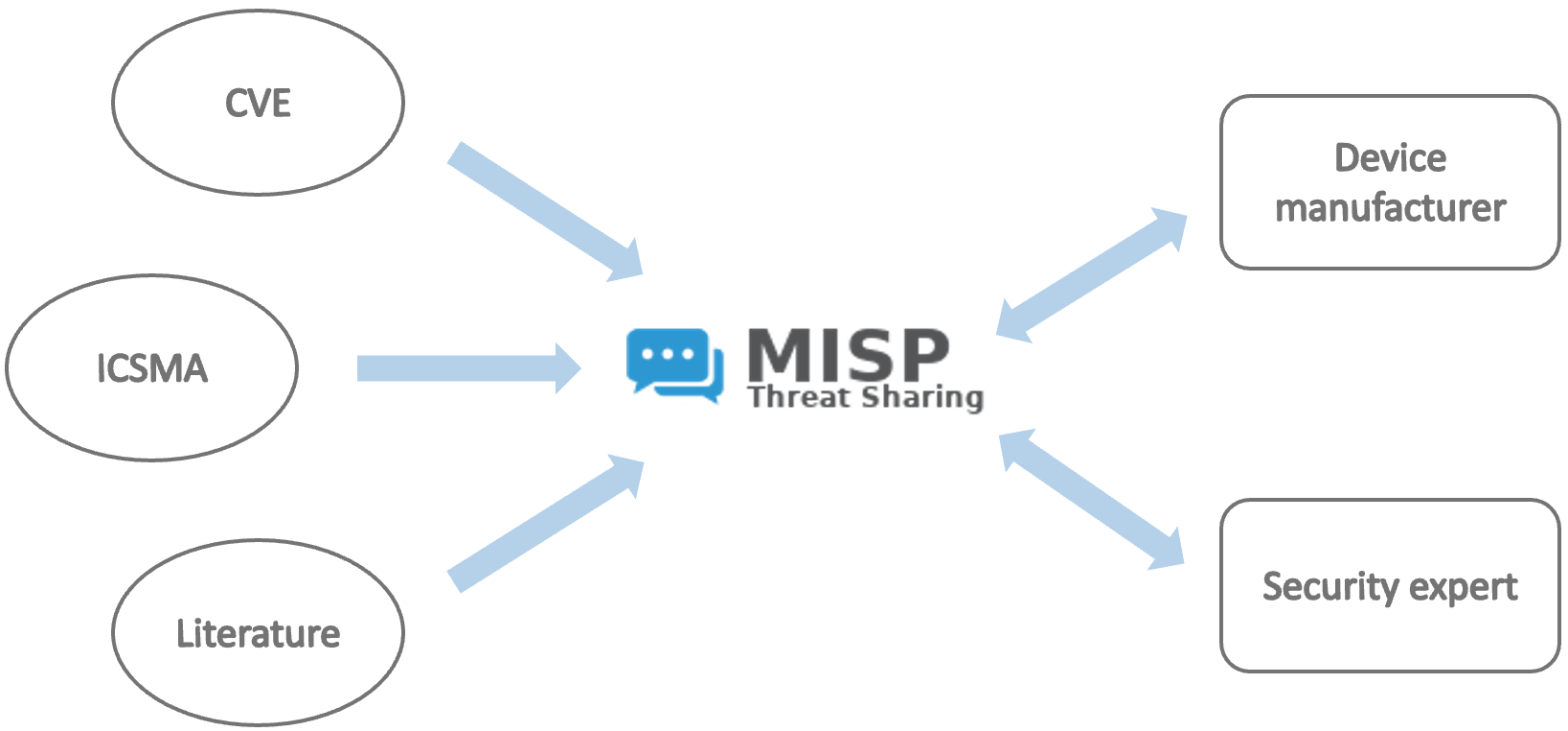
CTIS Architecture: Information sourced from CVE, ICSMA, and the scientific literature (**left**) feeds into the MISP platform (**center**), which organizes and processes the data for use by relevant entities such as device manufacturers and security experts (**right**).

Our solution leverages MISP to develop a Cyber Threat Information Sharing (CTIS) platform specifically tailored for medical devices, which we call *MMISP*. This platform is designed to collect and consolidate information from the various sources mentioned in Section 3 to provide a unified data repository and facilitating seamless interaction among the different entities involved, as illustrated in Figure 2.

**Figure 3:**
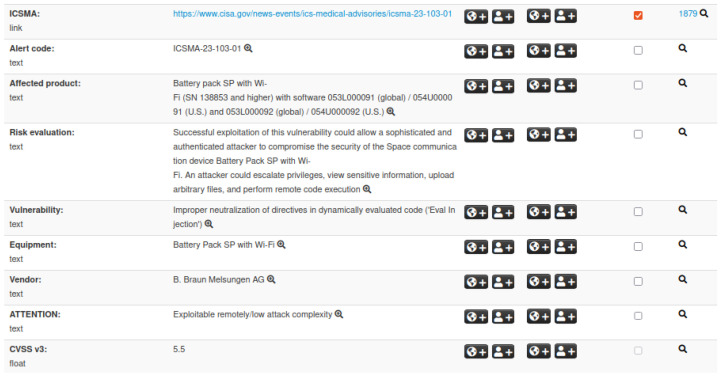
Illustration of an ICSMA event within the MISP platform. The visualization includes the external ICSMA reference, details on affected products, risk evaluation, vendor information, and related attributes.

In particular, we utilize two types of IoC events: *CVE* and *ICSMA*. To manage these event categories effectively, it is essential to define their respective structures. The MISP platform includes a default structure for IoC events related to CVEs; therefore, we only define a custom structure for ICSMA events. This structure incorporates all of the relevant information outlined in Section 3.2, including details on vulnerabilities and associated CVEs, affected medical devices, potential impacts, and recommended mitigations. Figure 3 presents an example of an ICSMA event, showcasing a subset of attributes within the defined structure.

Additionally, an effective vulnerability management strategy for medical devices must consider not only vulnerabilities that directly affect the device firmware or hardware but also those present in third-party libraries and components embedded within the device software. To this end, maintaining an up-to-date Software Bill of Materials (SBOM)—a detailed inventory of all software components, including libraries and dependencies—is essential.

An SBOM enables rapid identification of vulnerable components when new CVEs or ICSMAs are published, facilitating timely remediation. Moreover, initiatives and tools have emerged to automatically generate SBOMs from binary files, addressing situations where vendors do not provide this information.

Integrating SBOM management within MMISP would enhance its capability to track and correlate vulnerabilities across the entire software stack of medical devices, strengthening overall device security.

**Figure 4:**
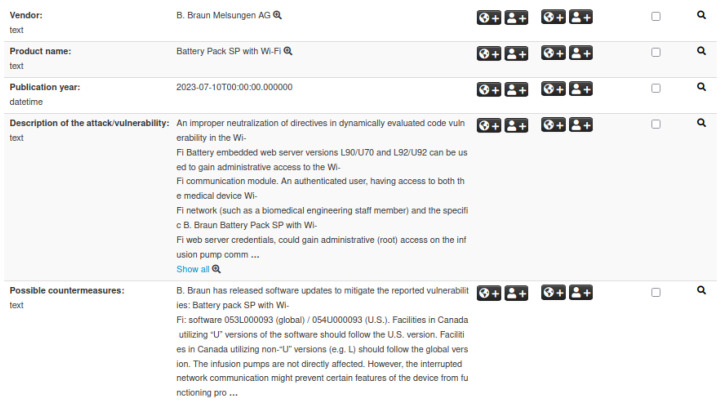
Example of a Medical Device event in MISP. The visualization highlights IoCs along with vendor, product name, vulnerabilities, and related attributes.

While Section 3.1 and Section 3.2 highlight the importance of IoC events for CVEs and ICSMAs, these two event types alone do not allow for an immediate identification of security issues related to specific categories of medical devices. To address this gap, we introduce a third dedicated structure that bridges the gap between these IoC events and medical device developers. This structure, referred to as *Medical Device*, is defined according to the taxonomy described in Section 4.1. It contains key information such as device and manufacturer details, known attacks and vulnerabilities, possible countermeasures, and associated ICSMAs. Figure 4 illustrates an example of a Medical Device event, displaying a selection of attributes that make up the corresponding structure.

### 5.1. Event Tag Taxonomy

To further enhance and extend the search capabilities for relevant information, a taxonomy has been defined to generate labels that can be associated as tags with Medical Device IoC events. This taxonomy is derived from a subset of the search criteria outlined in Section 4.1 and Section 4.2.

**Figure 5:**
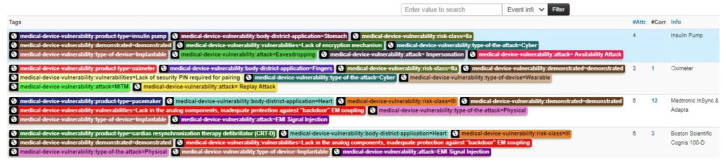
Example of MISP’s events list, showing four Medical Device events with associated tags. The ‘#Corr.’ column indicates the number of IoC events related to the selection.

By leveraging these tags, events can be filtered based on attributes such as risk class, potential attack vectors, or device type. This tagging system enables both visual and automated classification of events according to specific characteristics. An example of an event list on the MISP platform is shown in Figure 5, with some events associated with tags of different colors.

### 5.2. Event Correlation

An essential feature of the MISP platform is its ability to correlate IoC events based on specific attributes. The selection of correlatable attributes can be tailored to individual needs, allowing for a more focused or broader retrieval of related IoC events.

**Figure 6:**
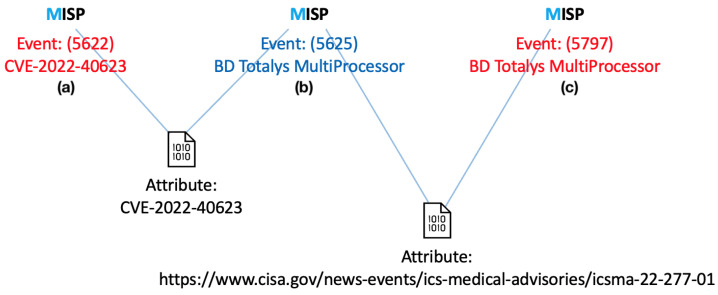
Example of IoC event correlation: First, a CVE event (**a**) is linked to an ICSMA event (**b**) via the ‘CVE ID’ attribute; then the ICSMA event is correlated with a Medical Device event (**c**) through the ‘ICSMA external link’ attribute (https://www.cisa.gov/news-events/ics-medical-advisories/icsma-22-277-01, accessed on 10 July 2025).

For instance, this correlation mechanism enables the retrieval of information on associated ICSMAs or CVEs starting from a specific Medical Device. Similarly, it allows identifying other Medical Devices linked to the same ICSMA or CVE. Figure 6 illustrates an example of correlation between a CVE, an ICSMA, and a Medical Device.

### 5.3. MISP Dashboard

**Figure 7:**
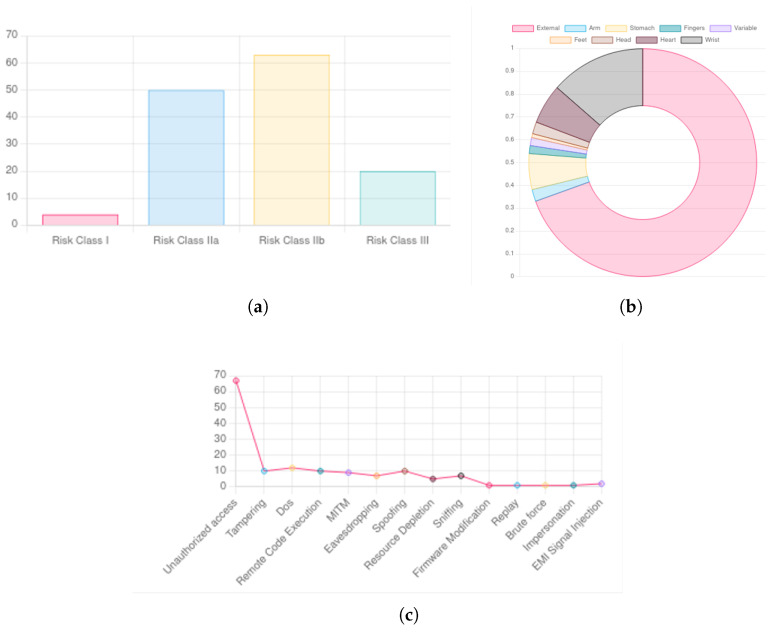
IoC events visualization: (**a**) risk class, (**b**) body district, (**c**) attack type.

The MISP platform provides a customizable “Dashboard” feature, allowing users to integrate widgets that highlight relevant information. Figure 7 presents an example where we have developed custom widgets to visually represent analyses conducted on IoC events within the system.

Specifically, Figure 7a illustrates the classification of IoC events based on the risk class of the associated medical devices. Figure 7b categorizes events according to the body regions where the affected devices are applied. Lastly, Figure 7c displays classifications based on the type of potential attacks.

The use of charts and other visualization techniques greatly enhances data interpretability within the platform. This added level of abstraction makes the information accessible to non-technical users while preserving the necessary technical depth.

## 6. MMISP Use Case

To illustrate how MMISP supports the security-by-design paradigm, we present the following use case. A development team is designing a new medical device for monitoring and collecting vital patient data. Given the critical importance of patient data security, the team aims to ensure that the device is resilient to cyber threats and compliant with security standards. The team leverages MMISP to access historical vulnerability data from similar medical devices and components already deployed in the field, enabling informed design decisions before the deployment of their own device. Specifically, they leverage MMISP to support the following steps:

- *Information Gathering*—The team utilizes MMISP to collect intelligence on cyber threats targeting medical devices. They focus on vulnerabilities (CVEs), products and components (CPEs), and industry-specific threats (ICSMA). For example, they identify vulnerabilities affecting widely used Bluetooth modules that are considered for integration.
- *Vulnerability Analysis*—MMISP allows the team to assess the technical details of discovered vulnerabilities, such as affected software versions and exploit availability. In one instance, a vulnerability affecting a third-party communication library prompts them to reconsider component selection.
- *Risk Assessment*—Using MMISP, the team evaluates the risks associated with each identified vulnerability, considering factors such as exploitability and potential impact.
- *Integration into the Development Process*—The team applies security insights from MMISP to strengthen their device’s design. This may involve patching vulnerabilities, isolating critical components, or implementing robust authentication mechanisms.
- *Continuous Monitoring*—Throughout the device’s lifecycle, the team relies on MMISP to stay informed about emerging threats and vulnerabilities, ensuring long-term security.
- *Regulatory Compliance*—The team leverages MMISP data to verify compliance with medical device security regulations and industry standards.

By proactively addressing cybersecurity risks, the development team enhances the safety and reliability of their medical device. This security-by-design approach safeguards both patient data and the integrity of the device’s operations.

## 7. Limitations and Future Directions

Although MMISP provides a promising framework for integrating threat intelligence into the secure development of medical devices, the current work should be considered a proof-of-concept rather than a validated system. At this stage, the platform has not yet been deployed or tested in real-world development pipelines, and no quantitative evaluation has been conducted to measure its actual impact.

As such, one key limitation of this study is the lack of empirical validation. The examples and use cases provided are hypothetical and illustrative, and are intended to demonstrate the potential applications of MMISP rather than serve as evidence of its effectiveness. Future work should focus on designing pilot studies with medical device manufacturers where MMISP is integrated into development workflows and assessed through measurable indicators such as time-to-detection of vulnerabilities, threat coverage, and design improvement metrics. To address these aspects, future studies should: (i) validate MMISP in controlled environments through case studies and testing with development teams, and (ii) investigate the adoption of machine learning models for automated threat classification and prioritization.

Furthermore, the effectiveness of MMISP heavily relies on the establishment of a robust network of participating nodes that actively engage with the platform. It is critical that these nodes do not merely act as passive sources of information but also contribute by sharing their own vulnerability data and threat intelligence. By becoming active sources themselves, these participants can enrich the collective knowledge base, fostering a collaborative ecosystem that enhances the overall security posture of medical device development. However, incentivizing such active participation requires addressing potential barriers, including trust issues, data privacy concerns, and the development of standardized protocols for secure information sharing.

Additionally, scalability challenges must be considered as the number of participants in the network grows significantly. A substantial increase in active nodes could strain the platform’s infrastructure, leading to potential bottlenecks in data processing, storage, and real-time analysis. Issues such as latency in threat information dissemination, data duplication, and the management of conflicting reports may arise, potentially undermining the platform’s reliability. Future research should explore distributed architectures, load balancing mechanisms, and efficient data validation techniques to ensure that MMISP remains effective and responsive even at scale.

## 8. Conclusions

In this paper, we have emphasized the importance of adopting a security-by-design approach in the development of medical devices. We have analyzed the most important sources of both certified and non-certified information, highlighting the challenges posed by the heterogeneity of content and data representation. This variability presents a significant barrier to gaining comprehensive knowledge of the security risks associated with these devices.

The MISP platform offers a valuable solution by acting as both an aggregator and an enhancer of cybersecurity information. We have demonstrated how it can be leveraged for CTIS in the medical device domain by defining specialized IoC event structures and taxonomies. One of MISP’s most powerful features—its ability to correlate events—is particularly beneficial in this context, facilitating deeper analysis and stronger security insights.

Furthermore, we have illustrated how the MISP platform can support various stages of a medical device’s lifecycle, from risk assessment to continuous monitoring and regulatory compliance. In conclusion, we firmly believe that a dedicated MISP instance for medical devices can serve as a crucial tool for research, threat analysis, and the dissemination of security-related information, ultimately enhancing the protection of these critical healthcare technologies.

## Figures and Tables

**Table 1:** Taxonomy used to classify vulnerable medical devices.

Criterion	Description
*Body District*	Anatomical region where the device is applied (e.g., heart,
	stomach, brain).
*Product Type*	Functional category of the device (e.g., insulin pump, pacemaker).
*Device Type*	Usage-based classification: wearable, implantable, on-site, etc.
*Year*	Year in which the vulnerability was discovered or reported.
*Attack Type*	Nature of attacks: cyber (e.g., network-based), physical, or hybrid.
*Vulnerability Type*	Type of security flaw (e.g., lack of encryption, default credentials).
*Vulnerability Severity*	CVSS score indicating the criticality of the vulnerability.
*Risk Class*	Regulatory risk classification (e.g., Class I, IIa, IIb, III).

## Data Availability

The raw data supporting the conclusions of this article will be made available by the authors on request.

## References

[1] Anderson R. A security policy model for clinical information systems. Proceedings of the 1996 IEEE Symposium on Security and Privacy.

[2] HIPAA (2025). Healthcare Data Breach Statistics. https://www.hipaajournal.com/healthcare-data-breach-statistics/.

[3] Health D. (2025). 120+ Latest Healthcare Cybersecurity Statistics for 2025. https://www.dialoghealth.com/post/healthcare-cybersecurity-statistics.

[4] Clusit (2024). Rapporto Clusit Healthcare 2024. https://clusit.it/blog/rapporto-clusit-healthcare-2024/.

[5] Panahi O. (2025). Secure IoT for healthcare. Eur. J. Innov. Stud. Sustain..

[6] Yaqoob T., Abbas H., Atiquzzaman M. (2019). Security vulnerabilities, attacks, countermeasures, and regulations of networked medical devices—A review. IEEE Commun. Surv. Tutor..

[7] Newaz A.I., Sikder A.K., Babun L., Uluagac A.S. Heka: A novel intrusion detection system for attacks to personal medical devices. Proceedings of the 2020 IEEE Conference on Communications and Network Security (CNS).

[8] Hassija V., Chamola V., Bajpai B.C., Zeadally S. (2021). Security issues in implantable medical devices: Fact or fiction?. Sustain. Cities Soc..

[9] Sethuraman S.C., Vijayakumar V., Walczak S. (2020). Cyber attacks on healthcare devices using unmanned aerial vehicles. J. Med. Syst..

[10] McGraw G. (2006). Software Security: Building Security In.

[11] Sametinger J., Rozenblit J., Lysecky R., Ott P. (2015). Security challenges for medical devices. Commun. ACM.

[12] Yeng P.K., Wolthusen S.D., Yang B. (2020). Comparative analysis of software development methodologies for security requirement analysis: Towards healthcare security practice. Inf. Syst..

[13] FDA (2023). Cybersecurity in Medical Devices: Quality System Considerations and Content of Premarket Submissions. https://www.fda.gov/media/119933/download.

[14] European Parliament and Council of the European Union (2017). Regulation (EU) 2017/745 of the European Parliament and of the Council of 5 April 2017 on medical devices, amending Directive 2001/83/EC, Regulation (EC) No 178/2002, and Regulation (EC) No 1223/2009 and repealing Council Directives 90/385/EEC and 93/42/EEC. Off. J. Eur. Union.

[15] European Parliament and Council of the European Union (2017). Regulation (EU) 2017/746 of the European Parliament and of the Council of 5 April 2017 on in vitro diagnostic medical devices and repealing Directive 98/79/EC and Commission Decision 2010/227/EU. Off. J. Eur. Union.

[16] European Commission (2022). Medical Devices: Guidance on Cybersecurity for Medical Devices.

[17] Medicines and Healthcare Products Regulatory Agency (2023). Medical Device Stand-Alone Software Including Apps (Including IVDMDs).

[18] Central Drugs Standard Control Organization (2016). Guidance Document on Common Submission Format for Registration of Medical Devices in India.

[19] He Y., Aliyu A., Evans M., Luo C. (2021). Health care cybersecurity challenges and solutions under the climate of COVID-19: Scoping review. J. Med. Internet Res..

[20] CISA (2024). Secure by Design. https://www.cisa.gov/securebydesign.

[21] Khan R.A., Khan S.U., Khan H.U., Ilyas M. (2022). Systematic literature review on security risks and its practices in secure software development. IEEE Access.

[22] Souppaya M., Scarfone K., Dodson D. (2022). Secure software development framework (ssdf) version 1.1. NIST Spec. Publ..

[23] OWASP Foundation (2019). OWASP Software Assurance Maturity Model (SAMM) v2.0. https://owasp.org/www-project-samm/.

[24] Howard M., Lipner S. (2006). The Security Development Lifecycle.

[25] (2011). Information Technology—Security Techniques—Application Security—Part 1: Overview and Concepts.

[26] Cima A. (2022). SOC 2® Reporting on an Examination of Controls at a Service Organization Relevant to Security, Availability, Processing Integrity, Confidentiality, or Privacy. https://www.aicpa-cima.com/cpe-learning/publication/soc-2-reporting-on-an-examination-of-controls-at-a-service-organization-relevant-to-security-availability-processing-integrity-confidentiality-or-privacy.

[27] (2022). Information Security, Cybersecurity and Privacy Protection—Information Security Management Systems—Requirements.

[28] Nisha S. (2025). Securing Life-Saving Devices: Challenges and Solutions in Medical Device Cybersecurity. Int. J. Trend Sci. Res. Dev..

[29] CERT (2020). EI CERT Coding Standards. https://wiki.sei.cmu.edu/confluence/display/seccode.

[30] CERT (2018). Top 10 Secure Coding Practices. https://wiki.sei.cmu.edu/confluence/display/seccode/Top+10+Secure+Coding+Practices.

[31] Open Web Application Security Project Foundation OWASP Secure Coding Practices-Quick Reference Guide. https://owasp.org/www-project-secure-coding-practices-quick-reference-guide/.

[32] Open Web Application Security Project Foundation OWASP Top 10. https://owasp.org/www-project-top-ten/.

[33] Martinovic I., Davies D., Frank M., Perito D., Ros T., Song D. On the Feasibility of Side-Channel Attacks with Brain-Computer Interfaces. Proceedings of the 21st USENIX Security Symposium (USENIX Security 12).

[34] Halevi T., Saxena N. On Pairing Constrained Wireless Devices Based on Secrecy of Auxiliary Channels: The Case of Acoustic Eavesdropping. Proceedings of the 17th ACM Conference on Computer and Communications Security, CCS ’10.

[35] Kune D.F., Backes J., Clark S.S., Kramer D., Reynolds M., Fu K., Kim Y., Xu W. Ghost Talk: Mitigating EMI Signal Injection Attacks Against Analog Sensors. Proceedings of the 2013 IEEE Symposium on Security and Privacy.

[36] Gattu N., Imtiaz Khan M.N., De A., Ghosh S. Power Side Channel Attack Analysis and Detection. Proceedings of the 2020 IEEE/ACM International Conference On Computer Aided Design (ICCAD).

[37] Giechaskiel I., Rasmussen K. (2020). Taxonomy and Challenges of Out-of-Band Signal Injection Attacks and Defenses. IEEE Commun. Surv. Tutor..

[38] CVE Program (2025). CVE Numbering Authorities (CNAs). https://www.cve.org/programorganization/cnas.

[39] CVE Program (2025). CVE Program Mission. https://www.cve.org/.

[40] (2025). Cybersecurity & Infrastructure Security Agency. https://www.cisa.gov/.

[41] University of Rome Tor Vergata (2023). Cyber4Health. https://cyber4health.uniroma2.it/.

[42] European Commission (2025). European Medical Device Nomenclature (EMDN). https://webgate.ec.europa.eu/dyna2/emdn/.

[43] European Commission (2025). European Database on Medical Devices (EUDAMED). https://ec.europa.eu/tools/eudamed/#/screen/home.

[44] MISP Project (2025). MISP Open Source Threat Intelligence Platform & Open Standards for Threat Information Sharing. https://www.misp-project.org/.

